# Specific effects of Ca^2+^ ions and molecular structure of β-lactoglobulin interfacial layers that drive macroscopic foam stability†
†Electronic supplementary information (ESI) available. See DOI: 10.1039/c6sm00636a



**DOI:** 10.1039/c6sm00636a

**Published:** 2016-06-13

**Authors:** Björn Braunschweig, Felix Schulze-Zachau, Eva Nagel, Kathrin Engelhardt, Stefan Stoyanov, Georgi Gochev, Khr. Khristov, Elena Mileva, Dotchi Exerowa, Reinhard Miller, Wolfgang Peukert

**Affiliations:** a Institute of Particle Technology (LFG) , Friedrich-Alexander-Universität Erlangen-Nürnberg (FAU) , Cauerstraße 4 , 91058 Erlangen , Germany . Email: bjoern.braunschweig@fau.de; b Cluster of Excellence Engineering of Advanced Materials (EAM) , Nägelsbachstr. 49b , 91052 Erlangen , Germany; c Erlangen Graduate School in Advanced Optical Technologies (SAOT) , Paul-Gordan-Straße 6 , 91052 Erlangen , Germany; d Interdisciplinary Center of Functional Particle Systems , Haberstraße 9a , 91058 Erlangen , Germany; e Institute of Physical Chemistry , Bulgarian Academy of Sciences , 1113 Sofia , Bulgaria; f Max-Planck-Institute of Colloids and Interfaces , 14476 Golm/Potsdam , Germany

## Abstract

β-Lactoglobulin (BLG) adsorption layers at air–water interfaces were studied *in situ* with vibrational sum-frequency generation (SFG), tensiometry, surface dilatational rheology and ellipsometry as a function of bulk Ca^2+^ concentration. The relation between the interfacial molecular structure of adsorbed BLG and the interactions with the supporting electrolyte is additionally addressed on higher length scales along the foam hierarchy – from the ubiquitous air–water interface through thin foam films to macroscopic foam. For concentrations <1 mM, a strong decrease in SFG intensity from O–H stretching bands and a slight increase in layer thickness and surface pressure are observed. A further increase in Ca^2+^ concentrations above 1 mM causes an apparent change in the polarity of aromatic C–H stretching vibrations from interfacial BLG which we associate to a charge reversal at the interface. Foam film measurements show formation of common black films at Ca^2+^ concentrations above 1 mM due to considerable decrease of the stabilizing electrostatic disjoining pressure. These observations also correlate with a minimum in macroscopic foam stability. For concentrations >30 mM Ca^2+^, micrographs of foam films show clear signatures of aggregates which tend to increase the stability of foam films. Here, the interfacial layers have a higher surface dilatational elasticity. In fact, macroscopic foams formed from BLG dilutions with high Ca^2+^ concentrations where aggregates and interfacial layers with higher elasticity are found, showed the highest stability with much smaller bubble sizes.

## Abstract 2

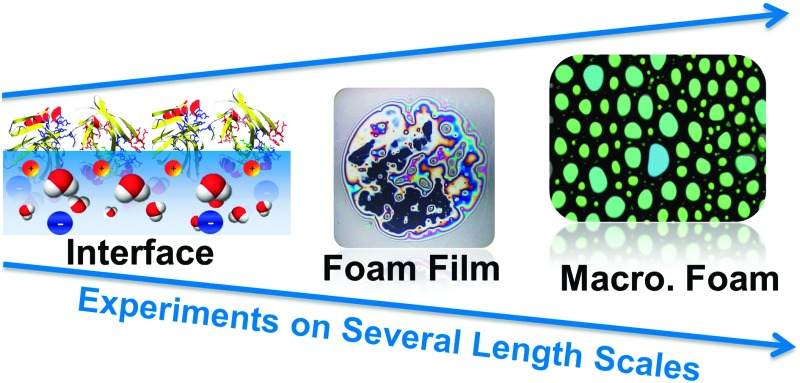
β-Lactoglobulin adsorption layers at air–water interfaces were studied with sum-frequency generation, tensiometry, surface dilatational rheology and ellipsometry.

## 1. Introduction

Air–water interfaces are ubiquitous in aqueous foams and for that reason both molecular structure and molecular interactions within the interfacial plane are a major driving force that influences bubble coalescence, Ostwald ripening and foam drainage.^1^ In each of these cases the interface can act as a potential barrier preventing or promoting the latter. Previous studies have shown that a combination of tensiometry and investigations of foam films as well as macroscopic foam can be extremely useful in determining correlations along the hierarchical chain of macroscopic foam.^2,3–6^ In particular, macroscopic properties of protein foams such as foam stability or foam rheology can be correlated to and are strongly dependent on the molecular structure and charging state of air–water interfaces which have been modified by surface active molecules such as proteins or surfactants.^7,8^ In fact, for bovine serum albumin (BSA) and β-lactoglobulin (BLG) it was shown that thick adsorbate layers of proteins at the interface with strongly reduced electrostatic intermolecular repulsion yielded the highest foam stability.^4,5,9^

Particularly for foams in dairy products the interactions of BLG, a major whey protein, and Ca^2+^ ions is important due to the high concentration of Ca (∼33 mM)^10^ in bovine milk which is naturally rich of calcium. While calcium binding to whey proteins is important, the latter does also play a major role in the cell metabolism^11^ where Ca^2+^ can have many functions such as co-factors for enzymes or as promoter for signal transduction. However, little molecular-level information is available on the effects of Ca^2+^ ions for charge regulation of protein surfaces at gas–water interfaces or the importance of Ca^2+^ for protein–protein interactions. For proteins in bulk solutions, previous studies indicate that a charge reversal can be caused by interaction with Ca^2+^ ions: Molina-Bolívar *et al.* ^12^ determined the *ζ*-potential of protein-covered latex particles and showed that it could be tuned from negative to positive values at a concentration of ∼50 mM CaCl_2_. The authors attribute the polarity change of the *ζ*-potential to a specific ion effect of Ca^2+^ that is not seen in NaCl solutions. Evidence that Ca^2+^ can interact with BLG's carboxylates comes from a study by Simons *et al.* ^13^ where the authors show that the concentration of Ca^2+^ needed to increase thermal aggregation scaled with the number of available carboxylate groups which were systematically varied using modified BLG proteins. In addition, improved thermal aggregation was not found for monovalent salts indicating that Ca^2+^ effects are ion specific.^13^ In particular for BLG in the bulk solution, Zittle *et al.* ^14^ report that in the pH range of 6 to 8 binding of Ca^2+^ ions is equivalent to the net charge. Consequently, it is possible to use the interaction of Ca^2+^ with BLG to efficiently screen the protein's net charge at mildly basic pH values or even cause a charge reversal. Furthermore, Beierlein *et al.* ^15^ applied molecular dynamics (MD) simulations of bulk BLG proteins and reported that Li^+^, Na^+^, and K^+^ cations can form contact-ion pairs (CIPs) with Asp and Glu carboxylates at the BLG surface. In the case of Li^+^ metal-cations, MD simulations show a charge reversal within the first solvation shell at 100 mM LiCl.

In this article we address the question if interfacial properties of BLG and adsorbate induced charging of air–water interfaces can be changed in a way that affects also macroscopic foam stability *via* structure–property relations from the interface to the macroscopic foam. In addition, addressing the solvation of proteins at interfaces is not only important for foam formulation but does have also larger implications as it is equally important in colloid science and protein purification.

Using a combination of experimental techniques on several length scales from asymmetric air–water interfaces, foam films (symmetric air/liquid/air interfaces) and macroscopic foam we provide a coherent understanding on BLG stabilized aqueous foam and the effects of Ca^2+^.

## 2. Materials and methods

### 2.1 Sample preparation

β-Lactoglobulin was isolated as described previously^16^ and kindly provided by the group of Ulrich Kulozik (TU München, Germany). 15 μM BLG dilutions were prepared by dissolving the protein powder in ultrapure water (18.2 MΩ cm; total oxidizable carbon <5 ppb) which resulted in a pH of ∼6.7 in the absence of CaCl_2_. Subsequently, the ionic strength was increased by adding CaCl_2_ (Alfa Aesar, Puratronic). All samples were allowed to react and to equilibrate at least 30 min before the measurements were started. Addition of CaCl_2_ resulted in a decrease in pH to ∼6.2 and 6.1 for 10 and 100 mM CaCl_2_, respectively. In order to ensure the cleanliness of the necessary glassware, all parts which came in contact with the protein solutions were soaked in concentrated sulphuric acid with NOCHROMIX for at least 12 h and were thoroughly rinsed with ultrapure water subsequently. All experiments were carried out at 297 K room temperature.

### 2.2 *ζ*-Potential measurements

*ζ*-Potentials from 15 μM BLG solutions were measured with a Malvern Instruments Zetasizer Nano ZS. For each Ca^2+^ concentration at least five measurements with different cuvettes were performed and the results were averaged.

### 2.3 Ellipsometry

Thickness (*h*_1_) determination of BLG adsorption layers was performed with a phase modulated ellipsometer (Beaglehole Instruments Picometer) at a wavelength of 632.8 nm. For each experiment 15 μM BLG sample solution was poured into a Petri dish with a diameter of 10 cm. Before measurements were started the surfaces were equilibrated for ∼30 min. Subsequently, angle scans between 51° and 55° *vs.* the surface normal were performed in 0.5° steps. For every Ca^2+^ concentration, at least 5 measurements at different positions near the centre of the Petri dish were recorded and averaged. As reported previously for BSA and BLG adsorption layers,^4,5^ angle-resolved data from ellipsometry were fitted using a three-layer model with refractive indices (RI) of 1.33, 1.40 and 1.0 for the electrolyte sub-phase, the protein layer and the air phase, respectively. We point out that the accuracy of the absolute values for the layer thickness is determined by the adequateness of the assumed RI. The RI is, however, often *a priori* unknown. Nevertheless, for BLG adsorbate layers we have previously shown that the assumption of *n* = 1.40 provides thicknesses that are in good agreement with the layer thickness from neutron reflectivity measurements.^5,17,18^

### 2.4 Sum-frequency generation (SFG)

Vibrational SFG was performed with tuneable femtosecond IR pulses which had a bandwidth of >200 cm^–1^ and were mixed with an etalon filtered femtosecond pulse at 800 nm wavelength which had a bandwidth of <6 cm^–1^ after filtering. Further details on the laser system used to generate both pulses can be found elsewhere.^19^ The IR and the 800 nm beam were overlapped at the air–water interface and the reflected sum-frequency photons were detected with a combination of a spectrograph (Shamrock 303i) and an intensified CCD camera (Andor, iStar). SFG spectra in the range of C–H and O–H stretching vibrations were measured by tuning the frequency of the IR beam in steps of 150 nm. Depending on the signal strength, total acquisition times for a SFG spectrum in this region were between 4 and 8 min. SFG spectra were reproduced in two separate sets of experiments, each with a different experimenter (see ESI†). For materials with inversion symmetry, like in the present case, the sum-frequency photons are in dipole approximation only generated at the interface and the bulk is being ignored for symmetry reasons. The SFG intensity can be expressed as a function of resonant and weak non-resonant excitations of molecules at the interface and is consequently resonantly enhanced when the frequency of the IR pulse matches with the frequency of vibrational modes from interfacial molecules.^20–22^
1
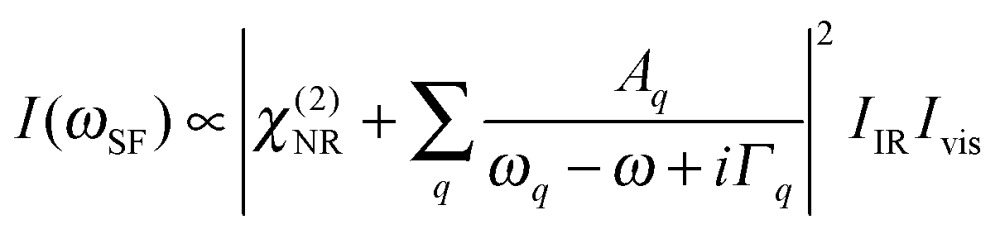
 The oscillator strength 
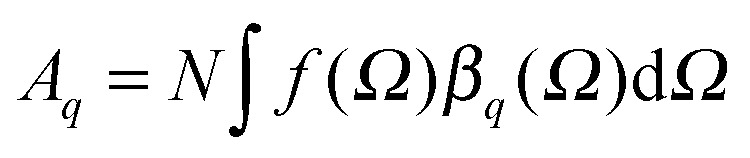
 of each SFG active vibrational mode is a function of the number density N of contributing molecules and their orientation distribution *f*(*Ω*) which is dependent on the solid angle *Ω*. For this reason, *f*(*Ω*) makes *A*_*q*_ directly dependent on the molecular order and orientation of interfacial molecules because random orientations – like in the isotropic bulk solution – would lead to negligible oscillator strengths while highly polar ordered interfacial molecules maximize SFG intensities of the associated vibrational bands. As already discussed and applied in previous studies of electrified interfaces, this effect offers the opportunity to probe electric field induced polar ordering and polarization at charged interfaces such as BLG modified air–water interfaces.^21–24^

### 2.5 Tensiometry and surface dilatational rheology

Drop shape analysis was performed with a pendent drop tensiometer (Krüss DSA100, Germany) using the oscillating drop module. The surface pressure *Π* = *γ*_0_ – *γ*, with *γ*_0_ being the surface tension of water (72.8 mN m^–1^) and *γ* the surface tension of an investigated solution, was first recorded as a function of time. After 30 min of adsorption, harmonic area oscillations (oscillation frequency of *f* = 0.1 Hz and area deformation of Δ*A*/*A* = 0.07) were applied and the surface tension response was recorded. These data allow for calculation of the values of the real *E*′ and imaginary *E*′′ parts of the complex dilatational visco-elasticity modulus. For the sake of simplicity, we will call *E*′ the surface dilatational elasticity and *E*′′ the surface dilatational viscosity.

### 2.6 Foam films

BLG foam films were studied in a Scheludko–Exerowa tube cell and the equivalent film thickness *h*_w_ was measured interferometrically^25^ as a function of the CaCl_2_ concentration. A foam film is formed in the middle of a biconcave drop of the measured solution. At equilibrium, the disjoining pressure which stabilizes the film is counterbalanced by the capillary pressure *P*_C_ [Pa] of the meniscus:^25^

2 *P*_C_ = 2*γ*/*R*

where *γ* [mN m^–1^] is the surface tension of the solution from which the film is formed and *R* [mm] is the radius of the meniscus curvature that can be approximated by the radius *R*_tube_ of the tube with well-wetted walls. In the present study, a cell with *R*_tube_ = 1 mm was used. All foam film experiments were performed with solutions of a slightly lower BLG concentration of 10 μM. After formation of the solution drop in the tube, it was allowed to rest for 30 min (saturation of the adsorption layers at film surfaces) and then a film was formed. The surface tension values in this setup and at this protein concentration (measured by tensiometry for the same time of adsorption) were found to decrease in the range from 52 to 48 mN m^–1^ with increasing Ca^2+^ concentration (compare to Fig. 3b, 72.8 mN m^–1^ – *Π*). Assuming an average value of *γ* = 50 ± 2 mN m^–1^ and eqn (2), the capillary pressure of the meniscus surrounding the film is determined by *P*_C_ = 100 ± 4 Pa. The measured equivalent thickness *h*_w_ of a foam film is larger than its real physical thickness *h*;^26^ the latter can be related to the equivalent thickness *via* a three-slab model accounting for the refractive index of the adsorption layers at the foam film surfaces:^25,26^

3 *h* = 2*h*_1_ + *h*_2_

**Fig. 1:**
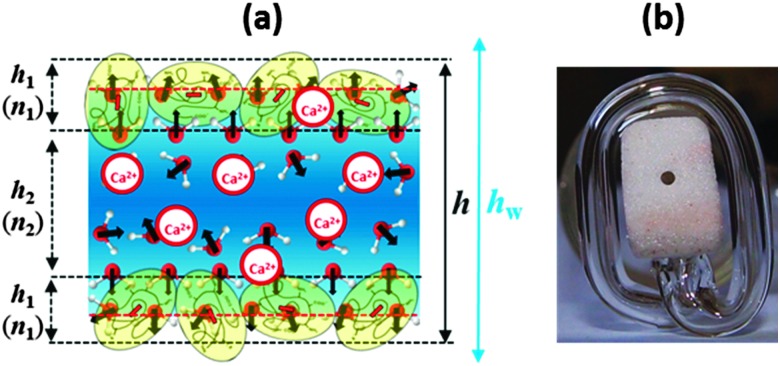
(a) Schematic representation of the structure of a BLG foam film (not to scale). Note that this scheme is only relevant for very low Ca^2+^ concentrations before significant charge screening and a possible charge reversal occurs at the interface; see also the discussion of Fig. 2. (b) Photograph of the modified porous plate cell with horizontal part of the capillary for experiments at low capillary pressures; the liquid from the porous plate is transferred into the horizontal part of the capillary and stays at the level of the film thus avoiding the action of a hydrostatic pressure.

4
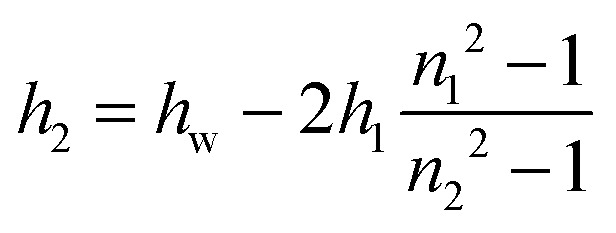
where *h*_1_ is the thickness of the adsorption layer at each of the film surfaces with a homogenous refractive index *n*_1_ and *h*_2_ is the thickness of the film liquid core with a homogenous refractive index *n*_2_ (see Fig. 1a). In the calculations of the equivalent film thickness *h*_w_^26^ and the real film thickness *h* (by eqn (3) and (4)) we used the same values *n*_1_ = 1.40 and *n*_2_ = 1.33 as for the analysis of the layer thickness from the ellipsometry experiments.

An Exerowa–Scheludko porous plate cell was used to measure the critical pressure of rupture *P*_cr_ of foam films.^25,26^ Foam films are formed in a small hole (radius of 0.5 mm) that is located in the center of a porous glass plate after the meniscus of a solution drop penetrates into the porous material. We used a modified version of this cell type with an elongated horizontal portion of the glass capillary (see Fig. 1b). This modification allows a higher precision for measurements at low capillary pressures of <100 Pa. This is particularly important for investigations during foam-film formation. The pressure in the glass chamber of the cell is controlled by a membrane pump and measured by a digital manometer with a sensitivity of ±1 Pa. The pressure applied onto the film surfaces is equal to the disjoining pressure *Π*_D_ in an equilibrium film with constant thickness. This allows for measuring *Π*_D_ *vs.* a thickness isotherm for the foam films under investigation. By this technique, it was possible to form films at pressures around 50–60 Pa and then to gradually increase the pressure until the film ruptures. For every studied solution, five films were formed sequentially and the pressure of rupture was measured. After film rupture, the solution drop was recovered in the hole of the porous plate and equilibrated again for another 30 min until the interfaces were saturated. Then the next foam film was formed. In this paper we present experimental data on foam film thickness *h* at constant pressure and the critical pressure of film rupture *P*_cr_.

### 2.7 Effects on the stability of macroscopic protein foams

Macroscopic foams were generated by purging ambient air with 0.3 l min^–1^ through a glass frit for 30 s. Subsequently to foam formation, foam decay was immediately measured. Foam analysis was performed with a DFA100 device (Krüss, Germany) allowing for measurements of the foam height and density over time with the help of image analysis and conductivity sensors along the foam column. Here, we define the parameter *S* about foam lifetime (stability) as *S* = 100 × *σ*_*t*_/*σ*_0_, where *σ*_*t*_ is the sum of conductivities measured at all sensors along the foam column at a given foam age *t* and *σ*_0_ is sum of the conductivities from all sensors along the foam column immediately after the gas flow was stopped. Using this definition of foam stability we include changes in the liquid content that are caused by *e.g.* drainage, foam structure and foam height. An analysis where we look at the foam height only is given in Fig. S4 in the ESI.†

All measurements were reproduced at least three times and the results were averaged. The structure of foam and the bubble size distributions were determined with the foam structure module of the DFA100.

## 3. Results

### 3.1 Structure and properties at air–water interfaces

**Fig. 2:**
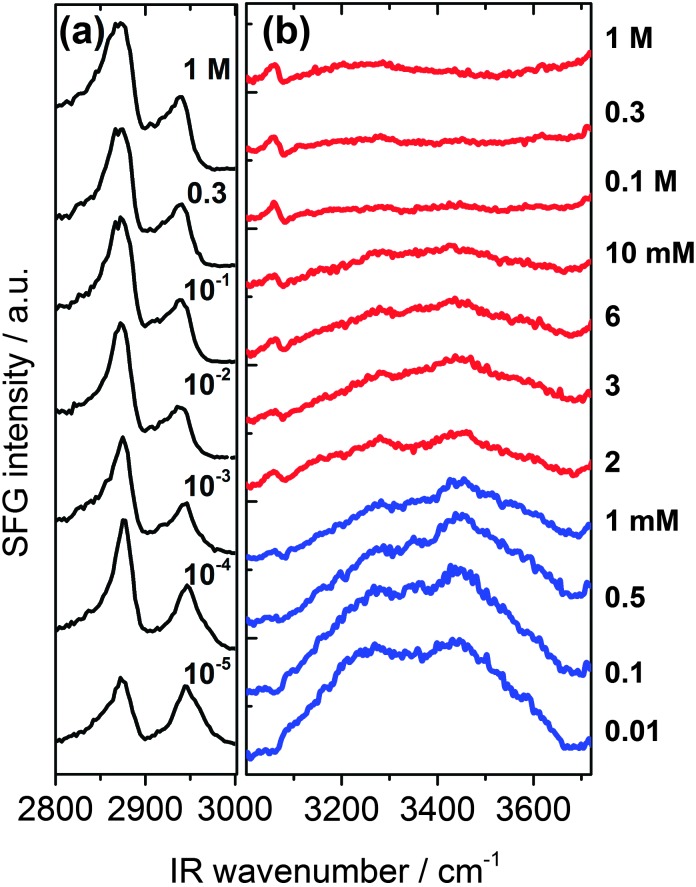
Vibrational SFG spectra of β-lactoglobulin (BLG) modified air–water interfaces as a function of Ca^2+^ concentration. (a) C–H stretching region that is dominated by CH_3_ bands originating from interfacial BLG proteins. (b) Shows the frequency region that is dominated by O–H stretching band from hydrogen bonded interfacial water molecules. Ca^2+^ concentrations for spectra were as indicated in (a) and (b).

In Fig. 2 we present SFG spectra of BLG modified air–water interfaces as a function of Ca^2+^ concentration (*C*_Ca^2+^_). The spectra are dominated by two narrow bands at 2878 and 2936 cm^–1^ which can be attributed to symmetric CH_3_ stretching vibrations and the CH_3_ Fermi resonance, respectively. Broad vibrational bands centered at 3200 and 3450 cm^–1^ are due to O–H stretching vibrations of interfacial water molecules.^21,22^ The broad distribution of bond angles and distances of hydrogen bonds at the interface gives rise to a broad distribution of O–H oscillator strengths which causes significant inhomogeneous broadening of O–H vibrational bands. Previously, the low frequency branch at 3200 cm^–1^ has been assigned to more tetrahedrally coordinated water molecules while the high frequency branch at 3450 cm^–1^ was assigned to interfacial H_2_O molecules in a more disordered environment.^21,22^ The depression at ∼3300 cm^–1^ is caused by the Fermi resonance of the bending mode of interfacial H_2_O molecules at 1650 cm^–1^.^27^

**Fig. 3:**
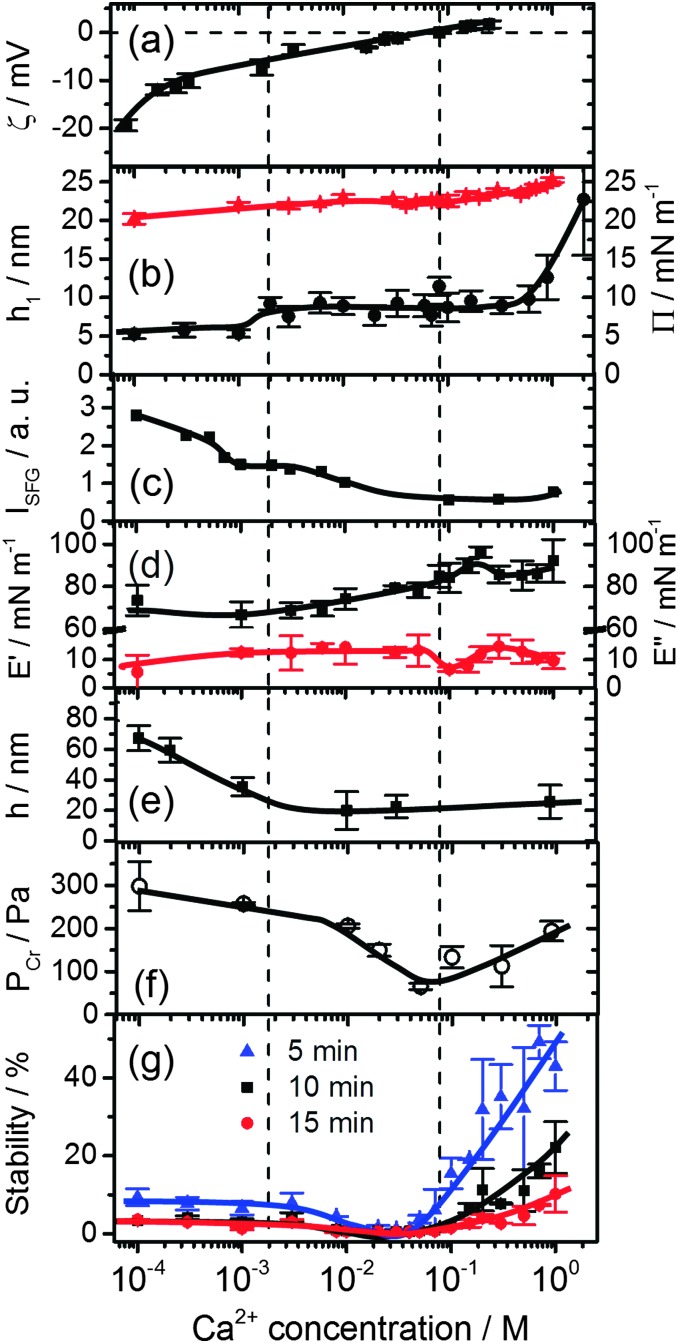
Effects of Ca^2+^ concentration on (a) the bulk *ζ*-potential and (b) layer thickness *h*_1_ (black circles) of β-lactoglobulin (BLG) modified at air–water interfaces as measured by ellipsometry and surface pressure *Π* (red triangles). (c) Intensity of O–H stretching band at 3200 cm^–1^ from SFG spectra in Fig. 2b. (d) Surface dilatational elasticity *E*′ (black squares) and viscosity *E*′′ (red circles). (e) Foam film thickness *h* at a capillary pressure *P*_C_ of (100 ± 4) Pa. (f) Critical pressure of film rupture *P*_cr_ and (g) stability of macroscopic foams from aqueous 15 μM solutions of BLG as a function of Ca^2+^ concentration and foam age. Lines are a guide to the eye.

A close inspection of Fig. 2 and 3c reveals a dramatic effect of Ca^2+^ concentrations on the SFG intensity of O–H stretching vibrations. At low concentrations (<2 mM) a substantial decrease in the intensity with increasing *C*_Ca^2+^_ is observed, while in the concentration range between 2 and 10 mM, the O–H intensity remains at a local plateau. Further increase in *C*_Ca^2+^_ above 10 mM leads first to a decrease in O–H intensity that is followed by a weak but noticeable recovery beyond 100 mM Ca^2+^. While O–H vibrational bands are strongly dependent of *C*_Ca^2+^_, contributions from C–H vibrational bands are for all concentrations very similar (Fig. 2a). Small changes in relative intensities of the contributing CH_3_ bands are actually caused by interferences with the O–H bands and their variations with *C*_Ca^2+^_. Obviously the net orientation of the contributing CH_3_ groups is not impaired by the Ca^2+^ induced increase in surface pressure and layer thickness (both Fig. 3b). This can only be explained when the inclusion of additional BLG proteins to the adsorbate layer occurs in a highly disordered state. In this state, the orientational average and thus the contribution of BLG molecules to the SFG spectra is negligible (see eqn (1) and discussion of *A*_*q*_). In previous studies where the O–H intensity and the charging state of the interfaces were controlled by the bulk pH, aggregate formation at the interface was found while little changes in intensity of the C–H bands were seen.^24,28^ As aggregates can be one possibility for the above observations, we will address this aspect in more detail below.

In this work, we have chosen a pH where the BLG molecules in the bulk solution carry a high negative net charge which leads to a *ζ*-potential of bulk BLG molecules close to –20 mV in the absence of Ca^2+^ (Fig. 3a). Obviously, the adsorption of BLG causes a macroscopic electric field at the air–water interface which can lead to polarized and ordered interfacial water molecules at high electric field strengths.^6,15,21,24,29^ As we have previously shown, SFG spectroscopy of BLG interfacial layers can be applied to determine relative changes in the interfacial electric field.^5,24^ In fact, the oscillator strengths *A*_*q*_ of O–H stretching bands and their phases can be used as highly sensitive markers for the charging state of electrified air–water interfaces.^30^ As can be clearly identified from Fig. 3a, increasing *C*_Ca^2+^_ leads to a dramatic decrease in the *ζ*-potential of BLG in the bulk to nearly zero values at ∼100 mM Ca^2+^. Small positive *ζ*-potentials are observed for concentrations >100 mM Ca^2+^. Obviously, additions of Ca^2+^ ions to the protein solution effectively influence the surface electric potential of the BLG entities in the solution at their shear plane and cause charge screening as well as overcharging at high concentrations. Taking these bulk processes into account, it is not surprising that the O–H intensity decreases because of the very efficient screening effect of Ca^2+^ counterions on the interfacial electric field that is induced by the presence of charged BLG molecules.

In addition to the latter observation, an apparent change in polarity of aromatic C–H stretching vibrations at ∼3060 cm^–1^ for concentrations between 0.1 and 2 mM can be inferred from a close inspection of Fig. 2b. This is likely due to a change in the phase of O–H vibrational bands caused by a charge reversal at the interface. In fact, previous studies on the effect of pH^4,5,28^ and oppositely charged surfactants^6,24^ on the properties of BLG layers have shown that similar but much more pronounced changes in the shape of the aromatic C–H band can be observed when the net charge within the interfacial plane is reversed.

In that case, a local minimum in SFG intensity at ∼3060 cm^–1^ is transformed into a local maximum. The phase reversal of the O–H bands at the air–water interface, that can be associated with a reversal of the interfacial net charge, occurs at much lower Ca^2+^ concentrations (∼1 mM) compared to the concentration where the *ζ*-potential of BLG proteins in the bulk solution is negligible (∼100 mM). Since ion specific effects of Ca^2+^ with BLG were already suggested by previous work,^13^ our results indicate that effects of Ca^2+^ at the interface are much more pronounced than in the bulk and lead, consequently, to an interfacial charge reversal at lower Ca^2+^ concentrations (∼1 mM). Further support for the strong interaction of calcium with BLG comes from an early study by Zittle *et al.* ^14^ who have applied analytic ultracentrifugation to determine the binding of calcium to native and denatured β-lactoglobulin. In fact, the study by Zittle *et al.* and also other studies^31^ show that within the pH range 6–7.2, a BLG molecule is capable to bind several Ca^2+^ ions.

Additional measurements of the thickness of the adsorption layers *h*_1_, the surface pressure and the surface dilatational rheology characteristics provide together with the SFG results a more complete picture of the molecular structure and interfacial properties which serves as a base to analyse the stability of foams and foam films as their building blocks. The layer thickness is presented in Fig. 3b. At concentrations <1 mM, a layer thickness *h*_1_ = 5.5 ± 0.6 nm is established. Such a value is compared to the thickness of BLG layers in the absence of Ca^2+^ already by a factor of two thicker than what was reported previously by ellipsometry^4,24^ and neutron reflectivity.^18^ This suggests that already at low Ca^2+^ concentrations BLG adsorption and possibly also aggregation at the air–water interface is promoted. However, the relative constant layer thickness *h*_1_ at low concentrations is accompanied by a gradual increase of both the surface pressure (Fig. 3b) and the surface dilatational elasticity *E*′ (Fig. 3d) which accounts for a compression of the surface layer and more intense intermolecular interactions that are responsible for the visco-elastic behaviour of the layer. A further increase in Ca^2+^ concentrations beyond ∼1.5 mM causes a jump to a second plateau of the layer thickness to *h*_1_ = 8.8 ± 1.3 nm. At concentrations >0.1 M where the bulk *ζ*-potential changes from negative to positive values (Fig. 3a), the most prominent changes in *E*′ and *E*′′ (Fig. 3d) occur. At 0.1 M the surface dilatational viscosity reaches a pronounced local minimum while the elasticity runs into a local maximum. At Ca^2+^ concentrations >0.3 M substantial increase in layer thickness *h*_1_ is observed from ∼9 to ∼23 nm at 1 M concentration. Comparing these changes with the O–H intensity which has decreased to nearly zero values at 0.1 M and which is dominated by electrostatic and orientation effects (see Section 2.4), it is reasonable to assume that the above changes must be caused by a change in intermolecular interactions within the surface layer which are dominated for concentrations <0.1 M by electrostatic interactions while for concentrations above 0.1 M the interfacial electric field is fully screened and van der Waals as well as hydrophobic forces can become dominant and consequently change the surface rheology of the layer. As we will show below, the molecular structure and changes in the magnitude and the type of the interactions have important consequences for the stability and thickness of foam films and the stability and structure of macroscopic foam.

### 3.2 Effects of Ca^2+^ on the properties of BLG foam films

Previous studies of foam films obtained from BLG solutions with different pH revealed that the film stabilization originates from the action of different types of repulsive surface forces such as electrostatic and/or short-range (steric) repulsion.^32^ The electrostatic disjoining pressure *Π*_EL_ of the films arises as a result of the interaction between the diffuse parts of the electrical double layers at each of the two film surfaces and depends simultaneously on both the magnitude of the electric potential within the interfacial double layer and the foam film thickness.^25^

The double layer potential is screened by increasing the ionic strength of the supporting electrolyte.^25^ This is also valid for the case of BLG foam films at a pH that is different from the isoelectric point pI, where an increase in ionic strength leads to a decrease in foam film thickness.^32^

**Fig. 4:**
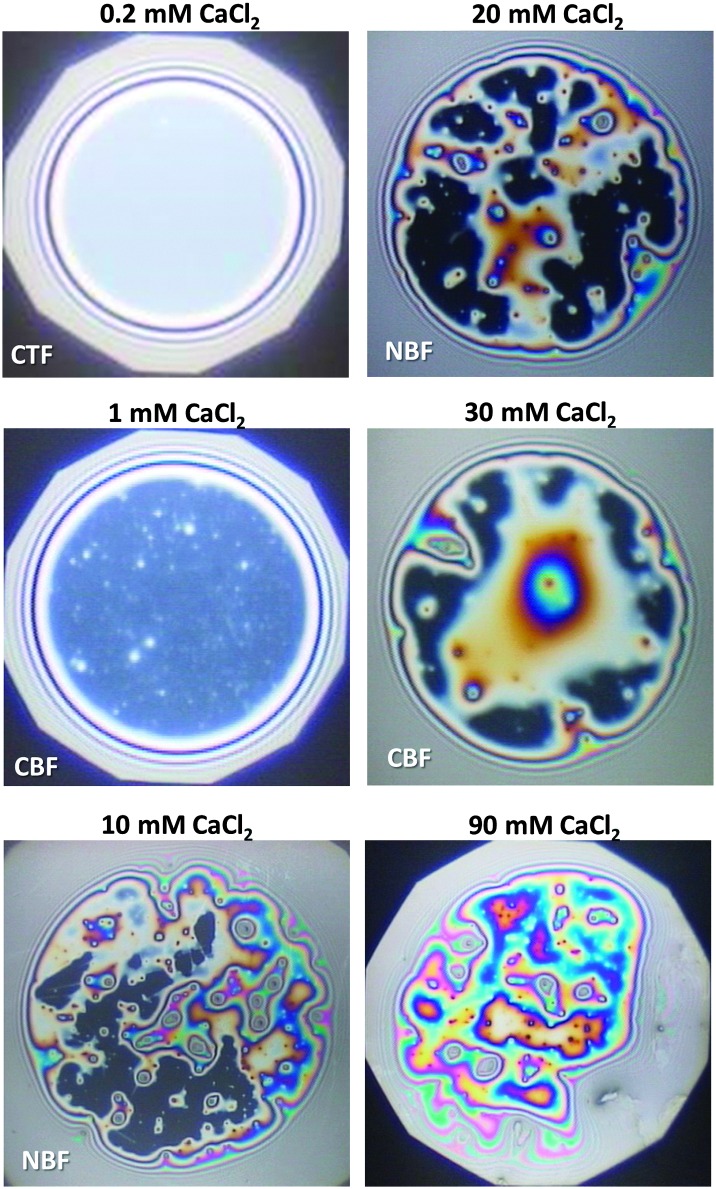
Images of BLG foam films for different Ca^2+^ concentrations; CTF – common thin film and CBF – common black film. The films were obtained in the tube cell at a capillary pressure *P*_C_ of 100 ± 4 Pa.

Let us now consider the thickness of BLG foam films as a function of Ca^2+^ concentrations in the tube cell. As discussed in the previous section, the interfacial BLG layers are negatively charged at the pH (>6) of the solutions that were used in our study, at least for *C*_Ca^2+^_ < 1 mM. Fig. 3e shows that initially the film thickness gradually decreases due to the screening effect of the increasing concentration of Ca^2+^ counterions. In the range of *C*_Ca^2+^_ between 0.05 and 0.2 mM, common thin films (CTF) are obtained as their equilibrium thickness is homogeneous (see Fig. 4) and comparatively large from 60 to 90 nm (Fig. 3e). This range of film thicknesses is attributable to weak electrostatic screening by these electrolyte concentrations. CTFs consist of two adsorption layers each with thickness *h*_1_ = 5.5 ± 0.6 nm (see the left-hand-side plateau in Fig. 3b) and a large solution core. The real film thicknesses *h* (Fig. 1a), presented in Fig. 3e, were calculated according to eqn (3) and (4) from the measured equivalent thicknesses *h*_w_ of the foam films. Here we have assumed a refractive index of the film core that is identical to the refractive index of neat water (*n*_2_ = 1.33) and a thickness of the adsorption layer *h*_1_ that is equal to 5.5 nm. Note that this value was previously determined from our ellipsometric measurements at the air–water interface for these concentrations (Fig. 3b).

For 1 mM bulk solutions, common black films (CBF)^25^ were obtained with thicknesses *h* of 35 ± 5 nm. Apparently, at this concentration the electrostatic disjoining pressure dominates the film thickness with a thickness of the electrolyte core *h*_2_ (Fig. 1a) of 24 ± 5 nm. The microscope image of this CBF is shown in Fig. 4. However, inhomogeneities in the film thickness are observed, which are indicative for the presence of protein aggregates confined between the two foam film surfaces. Obviously, these aggregates have sizes comparable to or larger than the liquid film core. In addition, at 1 mM we observe slower film drainage (not shown) than for the CTFs at *C*_Ca^2+^_ < 1 mM.

At *C*_Ca^2+^_ = 10 mM, the foam films drain slowly and exhibit island-like patterns due to the presence of larger aggregates that are confined within the film interior. Similar behaviour was reported in a study by Rullier *et al.* ^3^ where BLG aggregates have been produced and intentionally introduced into the bulk solutions. However, in our study, formation of black portions in the film is observed after a few minutes. These black areas have highly irregular shapes which are caused by the retarded film drainage and a comparatively high viscoelasticity of the film surfaces (Fig. 3d). Such portions of black film at 10 mM CaCl_2_ (Fig. 4) are identified as CBF with a thickness of about *h* = 20 ± 12 nm, which was calculated using eqn (3) and (4) from the measured equivalent film thickness *h*_w_ = 24 ± 10 nm and *h*_1_ = 8.8 ± 1.4 nm from ellipsometry (see the right-hand-side plateau in Fig. 3b). Note that the given value for *h* is an average over several measurements at different spots within the black portions of the foam film. The relatively large error is then related to inhomogeneities in the black film thickness. Foam films produced from BLG solutions with 30 and 900 mM CaCl_2_ were analysed in the same manner as for 10 mM and showed thicknesses in the black portions of *h* = 23 ± 4 nm and *h* = 26 ± 5 nm, respectively. We point out that the slight increase in *h* of the CBFs for *C*_Ca^2+^_ > 10 mM is comparable to the measurement error and is thus not necessarily associated with a real trend of a thickness increase.

For *C*_Ca^2+^_ between 40 and 100 mM, foam films with highly irregular film thicknesses were observed. For this reason a direct thickness measurement was not possible. The drainage of these films is blocked, which is consistent with rigid film surfaces and corresponds to the increase in surface dilatational elasticity *E*′ (Fig. 3d) and the nearly negligible *ζ*-potentials at ∼100 mM Ca^2+^. Surprisingly, this film behaviour changes with a further increase in Ca^2+^ concentrations, where again the formation of films with CBF portions is observed (see above).

The other parameter that we have used to characterise BLG foam films is the critical pressure of film rupture *P*_cr_ (Fig. 3f) which was studied in a modified porous plate cell. The highest stability of the foam films at concentrations <10 mM are attributable to the stabilizing effect of the electrostatic disjoining pressure *Π*_EL_^25,32^ and along with the decrease in film thickness, a reduction of the *Π*_EL_-magnitude leads to a decrease in foam film stability. However, a slight increase in *P*_cr_ was observed for 900 mM Ca^2+^ (Fig. 3f).

### 3.3 Structure and stability of macroscopic foam

We now address the effects of Ca^2+^ on the stability of macroscopic foam from 15 μM BLG solutions. Fig. 3g depicts the macroscopic foam stability as a function of Ca^2+^ concentration. Apparently a minimum in the foam stability is observed in the region between 20–50 mM Ca^2+^, which also correlates with the minimum in the foam film stability in Fig. 3f. Note that this minimum in foam stability is independent of the ‘stability’ definition – whether it is defined as above (see Section 2.7) or by simply looking at the loss in foam volume/height over time (Fig. S4 in ESI†). Thus, if *P*_cr_ is low, the macroscopic foam has low stability (lifetime).^33^ However, film stability increases only slightly after the minimum, while foam stability is markedly enhanced for *C*_Ca^2+^_ > 100 mM which will be addressed in the General discussion section below.

**Fig. 5:**
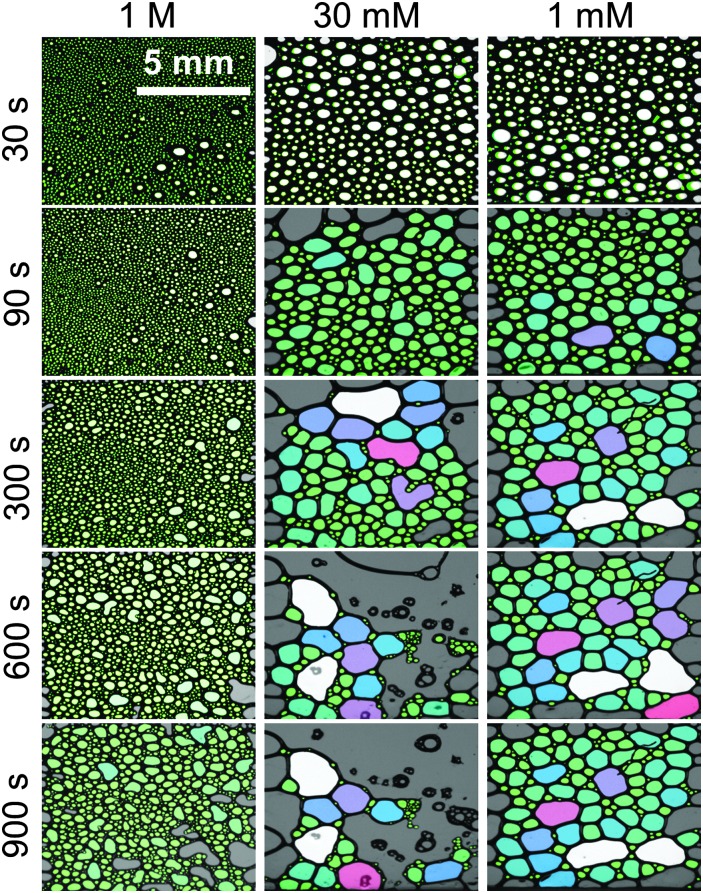
Structure and aging of macroscopic foam from 15 μM β-lactoglobulin solutions with 1 M, 30 mM and 1 mM CaCl_2_ concentrations. Foam age was as indicated, 30 s indicate that time after the gas flow was stopped. The lateral resolution was for images identical and is as indicated in the top left image. Bubbles are false colour coded for certain size fractions. Additional concentrations can be found in the ESI.†

The increase in macroscopic foam stability is accompanied by a change in foam structure as shown in Fig. 5 for three representative concentrations. Additional foam structures for concentrations of 10, 100 and 300 mM CaCl_2_ can be found in the ESI.† The initial structures at 1 and 30 mM are very similar, while for higher concentration, where much more stable foams are formed (Fig. 3e), the structure is drastically different. In fact, increasing the Ca^2+^ concentration leads to a substantial change in bubble size distribution: for *C*_Ca^2+^_ < 100 mM bubbles with a mean equivalent diameter of ∼80 μm and a large size fraction of bubbles between 250 and 500 μm is found directly after foaming (30 s in Fig. 5), while for *C*_Ca^2+^_ > 100 mM directly after foaming significantly smaller bubbles with a mean diameter of 9 μm are found with a much lower frequency of bubbles in the 250 to 500 μm size fraction (ESI†). Analysis of the changes in foam structure as function of foam age indicates that bubble coalescence and drainage are major destabilization pathways.

### 3.4 General discussion

Combining the experimental evidence from the results presented above we can now suggest a qualitative model of the interfacial charging state and structure which determines the observed changes in foam stability *via* structure–property relations:

By comparing the changes in the SFG spectra of BLG modified air–water interfaces and in the layer thickness *h*_1_ (Fig. 3b), we find that the first increase in layer thickness at 0.5 to 2 mM Ca^2+^ coincides with a steep decrease in O–H intensity that is accompanied by a change in polarity of the aromatic C–H band at 3060 cm^–1^ from a dip-like structure to a weakly pronounced maximum (Fig. 2b). As noted above similar changes were previously attributed to a change in the orientation of the mean electric field at the interface that is caused by the adsorbed BLG including their solvation shells.^4–6,15,24^

We now propose that the rapid decline in O–H intensity is caused by specific interaction of BLG with calcium ions. The strong interaction of Ca^2+^ with BLG molecules first rapidly screens the interfacial net charge (*C*_Ca^2+^_ < 1 mM) leading to conditions of a partial charge reversal and overcharging at high concentrations (*C*_Ca^2+^_ > 1 mM).

At this point, the question on possible binding sites and motives of Ca^2+^ at the BLG protein surface arises. Previous studies have shown that in many cases specific ion effects can be rationalized with a simple electrostatic model of ions and their affinity to bind water molecules – the so-called Law of Matching Water Affinities.^34^ Using this model, contact ion pair formation between Ca^2+^ and R–COO^–^ from amino acid side chains is likely because their water affinities are nearly matched. Allen and co-workers^35^ report in their SFG study of palmitic acid surfactants at air–water interfaces that complexes of Ca^2+^ ions with the carboxylate group can be formed. The authors concluded that at equilibrium conditions a mixture of bridging (2 Ca^2+^:1 R–COO^–^) and chelating (1 Ca^2+^:1 R–COO^–^) configurations is established for low concentrations of Ca^2+^ (0.1 M) while at 0.3 M concentrations only the bridging configuration is favoured. This means that at pH ∼ 6, binding of three Ca^2+^ ions to a BLG monomer are needed in order to neutralize the net charge of approximately –6*e*.^36,37^ For a further analysis we assume that at low concentrations Ca^2+^ binding occurs predominantly on carboxylate groups in bridging configurations. In addition, Tanford and co-workers^36^ report that 50 side chains with R–COO^–^ groups are accessible to Ca^2+^ ions. Because each of these groups can bind Ca^2+^ *via* contact ion pair formation, a calcium coverage of *θ* = 3/50 exists at conditions with a neutralized net charge (1–2 mM Ca^2+^). If we now apply a simple Langmuir adsorption model for the binding of Ca^2+^ 
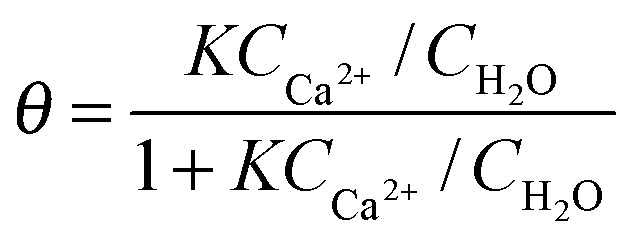
with *C*_Ca^2+^_ and *C*_H_2_O_ = 55.5 M being the concentrations of calcium and water, respectively, we can estimate the equilibrium constant *K.* This analysis, yields at *C*_Ca^2+^_ = 1.5 mM an equilibrium constant for Ca^2+^ binding to the hydrated carboxylates of BLG of log *K* ∼ 3.3 ± 0.2. This value is higher compared to previously reported^38^ equilibrium constants log *K* such as 1.43 for Glu amino acids, and 1.6 for Asp amino acids and ∼0.5 for acetic acid, but it is much lower compared to strongly calcium binding proteins such as parvalbumins (log *K* = 9.4). Based on our results we can calculate the Gibbs free energy for Ca^2+^ binding to hydrated carboxylate side chains on the protein surface of interfacial BLG to Δ*G* = –*RT* ln(*K*) = –18 ± 2 kJ mol^–1^.

Having established a picture for the molecular structure and electrostatic interactions of BLG interfacial layers as a function of *C*_Ca^2+^_, we now address the effects of Ca^2+^ on the structure and stability of foam films and macroscopic foam. As discussed in Section 3.2, the thickness of foam film strongly decreases with Ca^2+^ concentration until common black films with apparent aggregates are formed at 1 mM. Taking into account the results for the foam film thickness *h* in Fig. 3e, it becomes clear that electrostatic forces have been significantly screened between 1 and 10 mM Ca^2+^. This is fully consistent with our attribution of the spectral changes in vibrational SFG that point to a charge reversal at ∼1.5 mM. Furthermore, images of foam films in Fig. 4 indicate that for higher Ca^2+^ concentrations aggregates are formed and have in addition to the higher surface dilatational elasticity *E*′ (Fig. 3d) a stabilization effect on the foam films at >100 mM Ca^2+^. The presence of aggregates at the interface is consistent with the negligible change of C–H bands in our SFG spectra (Fig. 2) when the Ca^2+^ is increased while the surface pressure and layer thickness both increase simultaneously. As explained above, this is only possible if the additional molecules at higher Ca^2+^ concentrations have no net orientation which is the case when averaging over a larger number of aggregates at the interface. This stabilization due to aggregate formation in foam films and possibly also in the lamella of macroscopic foam, and the increase in *E*′ are obviously directly related to higher stabilities of macroscopic foam and partly to the origin of the smaller mean bubble sizes as compared to BLG foams from solution with *C*_Ca^2+^_ ≪ 100 mM. At this point we need to stress also the adsorption kinetics. At a bulk protein concentration of 15 μM, the adsorption of BLG to the interface is already fast (ESI†). Considering the kinetic data from surface pressure measurements (Fig. S5 in ESI†), we observed that increasing CaCl_2_ concentration remarkably enhances the rate of the adsorption kinetics (the slope d*Π*/d*t* increases) within the first few seconds (<5 s) of the adsorption process. Such time scale is comparable to that of the foam formation process and therefore, adsorption kinetics can be responsible for the observed smaller bubble sizes in the final foam at very high CaCl_2_ concentrations. For solutions aged more than *ca.* 1 min, comparison of the rate of the surface pressure change with time for solutions with 15 μM BLG and CaCl_2_ concentrations between 0 and 1 M reveals only slight acceleration of the rate of adsorption with an increase in CaCl_2_ concentration.

As we have shown above in Fig. 3a, also the bulk *ζ*-potential is fully screened at 100 mM and it is thus likely that also in the bulk solution BLG aggregates are formed to some extent. Their concentration is, however, relatively low as we could not find evidence for aggregates from dynamic light scattering (DLS). At this point it is interesting to discuss the study by Rullier *et al.* ^3,39^ on foams and foam films stabilized by BLG aggregates that were obtained by a thermal treatment of BLG solutions. These authors find that even for a very low quantity of aggregates (1%), the stability of macroscopic foam was substantially improved, while optical microscopy of foam films indicated the presence of aggregates as well. Rullier *et al.* ^40^ propose three different stabilization mechanisms that depend on the relative concentration of protein aggregates. It is proposed that at high concentrations of aggregated proteins, a gel-like network with non-aggregated proteins is formed while at intermediate concentrations aggregates can crosslink the two foam film surfaces and contribute to their stabilization in the process. The origin of the stabilization of foams with aggregate concentrations as low as 1% (at 1 g l^–1^ total concentration) is less clear from the latter study and is attributed to aggregates that are trapped inside the plateau borders in macroscopic foam where they can slow down drainage. Similar behaviour was recently reported for particle stabilized aqueous foams by Carl *et al.* ^41^

Obviously, the study by Rullier *et al.* ^40^ brings strong support to our conclusion that the increase in foam as well as foam film stability is driven by a combination of aggregated BLG molecules, that either form in the bulk solution at *C*_Ca^2+^_ ≫ 100 mM or predominantly at the interface at low concentrations <100 mM. In addition, Lexis and Willenbacher^8^ have proposed by comparing interfacial rheological properties with the rheology of macroscopic foam that BLG aggregates across foam lamellae can determine foam properties. The exact role of BLG aggregates at the air–water interface in the stabilization of the foam is, however, at this point unclear. Either the changes in molecular structure and lateral interactions at the air–water interface as discussed above are the only reasons, or these changes are additionally accompanied by a blocking effect where BLG agglomerates accumulate within the plateau borders and prevent or slow down foam drainage in the process.

## 4. Conclusions

Specific ion effects of Ca^2+^ on the molecular structure of β-lactoglobulin modified air–water interfaces and its influence on the stability of macroscopic foams from aqueous BLG solutions was studied on a molecular level. Vibrational sum-frequency generation (SFG) of the interfacial structure and charging state as well as complementary foam film measurements indicate a charge reversal at the air–water interface (at ∼1.5 mM Ca^2+^) from a negative net charge to a surface with a positive net charge. The charge reversal which is necessarily accompanied by a change of electrostatic intermolecular interactions drives β-lactoglobulin molecules at the interface into highly agglomerated but disordered layers with a maximum in layer thickness of ∼20 nm at 1 M CaCl_2_. In addition, at these concentrations the stability of macroscopic foam reaches a maximum. Obviously, foam properties are controlled by the surface molecular properties such as structure and intermolecular interactions of the adsorption layers *via* structure–property relations.
