# Supplementary material for: Specific effects of Ca2+ ions and molecular structure of β-lactoglobulin interfacial layers that drive macroscopic foam stability
Source: Soft Matter. 2016 Jun 13;12(27):5995–6004. doi: 10.1039/c6sm00636a (PMC5048339; doi:10.1039/c6sm00636a)
Supplement: Supplementary file 1 [file SM-012-C6SM00636A-s001.pdf]

Electronic Supplementary Material (ESI) for Soft Matter.
This journal is © The Royal Society of Chemistry 2016

Supporting Information

Specific Effects of Ca^{2+} Ions and Molecular Structure of β-Lactoglobulin Interfacial Layers that Drive Macroscopic Foam Stability

Björn Braunschweig,[a,b,c†] Felix Schulze-Zachau,[a,c] Eva Nagel [a], Kathrin Engelhardt,[a] Stefan Stoyanov [d], Georgi Gochev,[d,e] Khr. Khristov,[d] Elena Mileva,[d] Dotchi Exerowa,[d] Reinhard Miller,[e] and Wolfgang Peukert[a,b,c]

[a] Institute of Particle Technology (LFG), Friedrich-Alexander-Universität Erlangen-Nürnberg (FAU), Cauerstraße 4, 91058 Erlangen, Germany.

[b] Cluster of Excellence Engineering of Advanced Materials (EAM), Nägelsbachstr. 49b, 91052 Erlangen, Germany.

[c] Erlangen Graduate School in Advanced Optical Technologies (SAOT), Paul-Gordan-Straße 6, 91052 Erlangen, Germany

[d] Institute of Physical Chemistry, Bulgarian Academy of Sciences, 1113 Sofia, Bulgaria

[e] Max-Planck-Institute of Colloids and Interfaces, 14476 Golm/Potsdam, Germany

† email: bjoern.braunschweig@fau.de

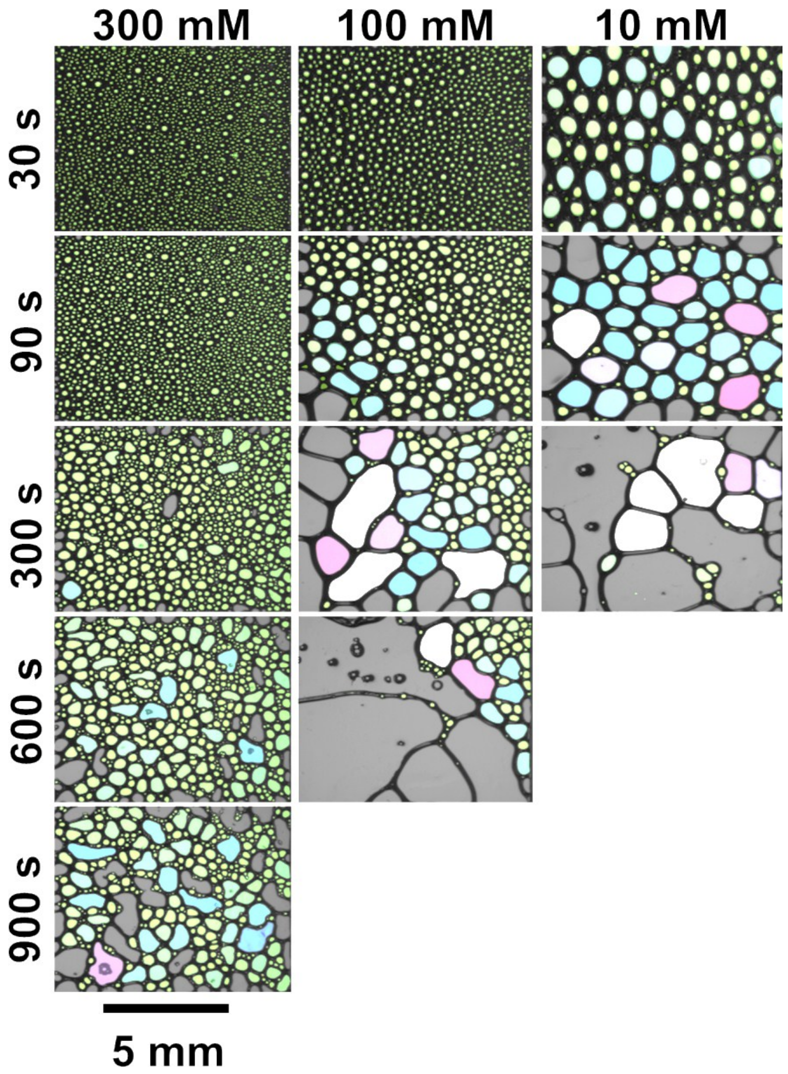

Figure S1 Structure and aging of macroscopic foam from 15 µM β-lactoglobulin solutions with 300, 100 and 10 mM $CaCl_2$ concentrations. Foam age was as indicated, 30 s indicate that time after the gas flow was stopped. The lateral resolution was for images identical and is as indicated in the top left image. Bubbles are false colour coded for certain size fractions.

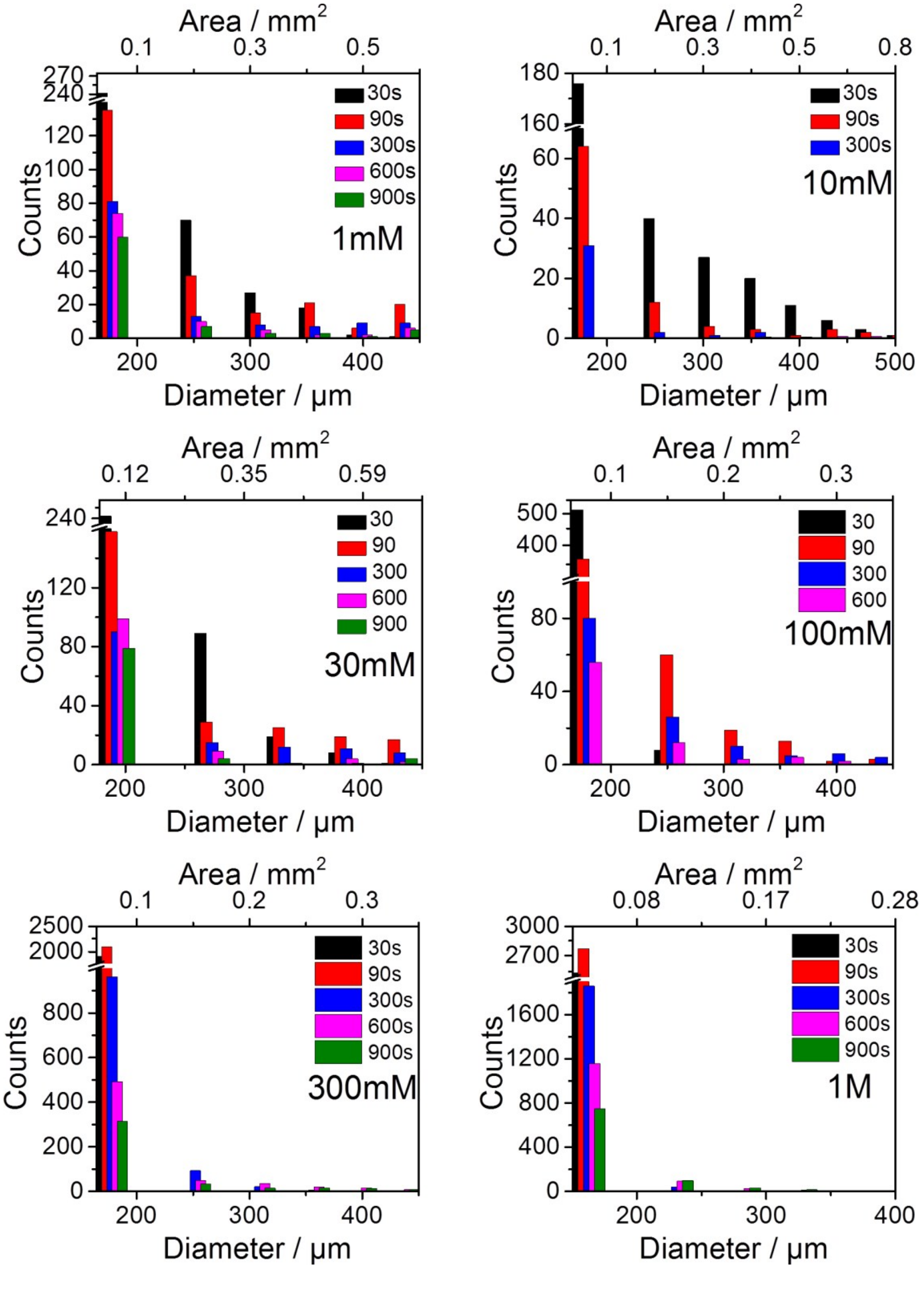

Figure S2 Bubble size distributions for macroscopic foam from 15 μM β-lactoglobulin solutions with different $CaCl_2$ concentrations and as a function of foam age as indicated in the Figure.

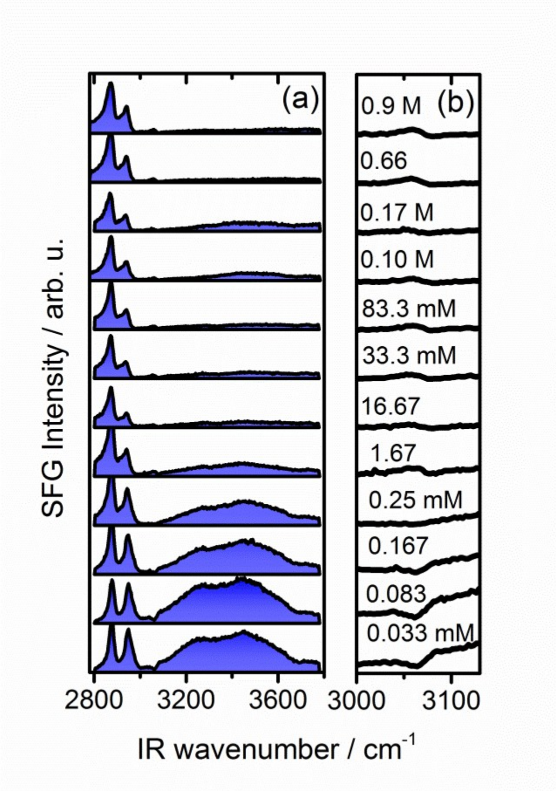

Figure S3. Second set of vibrational SFG spectra from β-lactoglobulin (BLG) modified air-water interfaces as a function of Ca^{2+} concentration. (a) shows the C-H and O-H stretching region (b) shows the region that is dominated by the aromatic C-H stretching mode. Note that we use the interference of this band with the O-H stretching bands to determine the polarity of the interfacial net charge. For further discussions see main text.

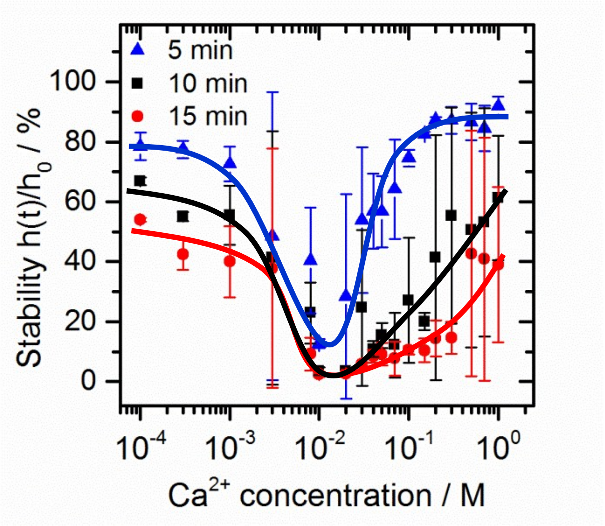

Figure S4. Foam stability of 15 μM β-lactoglobulin (BLG) solutions with different $CaCl_2$ concentrations and as a function of foam age as indicated in the Figure, determined by evaluation of foam heights h(t) at different times relative to the initial foam height h_0. Lines are a guide to the eye.

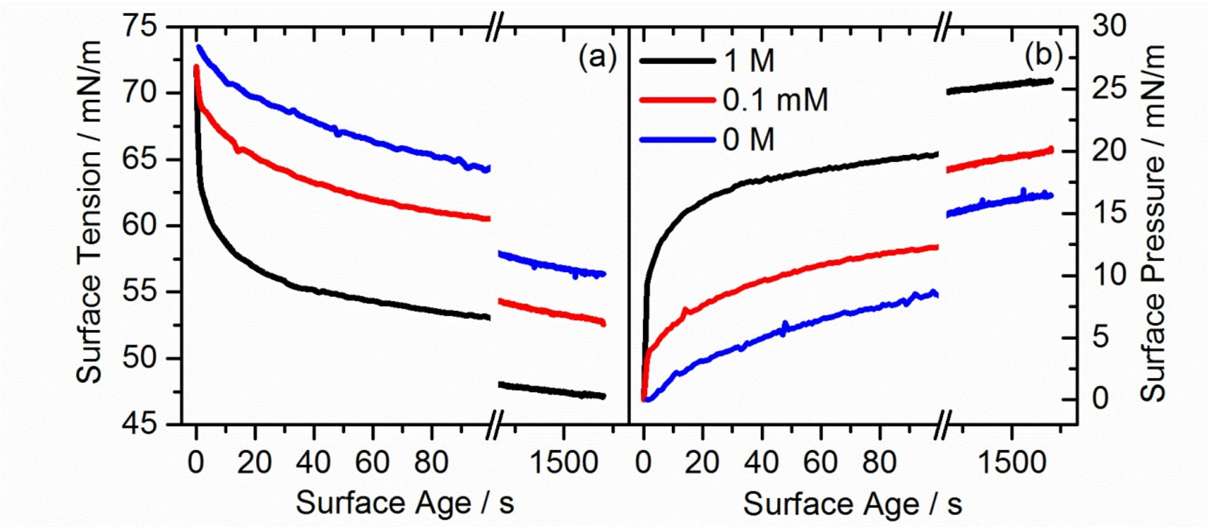

Figure S5. Surface tension (a) and surface pressure (b) of 15 μM β-lactoglobulin (BLG) solutions for different $CaCl_2$ concentrations as a function of surface age.
